# A Matching Game-Based Data Collection Algorithm with Mobile Collectors

**DOI:** 10.3390/s20051398

**Published:** 2020-03-04

**Authors:** Chun Zhang, Shumin Fei

**Affiliations:** 1 School of Computer Science, Nanjing University of Posts and Telecommunications, Nanjing 210023, China; 2 School of Automation, South and East University, Nanjing 210018, China

**Keywords:** wireless sensor networks, data collection, mobile collectors, matching game, data delay

## Abstract

Data collection is one of the key technologies in wireless sensor networks. Due to the limited battery resources of sensors, mobile collectors are introduced to collect data instead of multi-hop data relay. However, how to decrease the data delay based on the cooperation of mobile collectors is a main problem. To solve this problem, a matching game-based data collection algorithm is proposed. First, some high-level cluster heads are elected. Second, by introducing a matching game model, the data collection problem is modeled as a one to one matching problem. Then, according to the preferences of mobile collectors and cluster heads, the benefit matrices are established. Based on the proposed matching algorithm, each mobile collector selects a cluster head to collect the data packets. Performance analysis proves that the matching result is stable, optimal, and unique. Simulation results show that the proposed algorithm is superior to other existing approach in terms of the reduction in data delay.

## 1. Introduction

The wireless sensor network consists of a large number of sensor nodes. The sensor nodes monitor the environment, process the data packets and then send the data packets to other sensor nodes or the sink. With the development of computer network technology and sensor technology, wireless sensor networks are more and more widely used in various fields [1,2,3]. Whether the wireless sensor network is applied in which field, it is data-centric, that is, obtaining information as an important goal. The sink makes decisions based on the information obtained from the sensor nodes. Therefore, data collection is a key technology of a wireless sensor network.

Multi-hop transmission is commonly used in wireless sensor networks. Sensor nodes are divided into several clusters. Each cluster elects a cluster head. Ordinary nodes send information to the cluster head they are attached to. Cluster heads merge the information and send it to the sink. LEACH [4] algorithm is a classic cluster algorithm. First, at the beginning of each period, based on the number of cluster heads needed in the network and the number of times each node has been elected as the cluster head, some nodes are elected as cluster heads. Then other nodes decide which cluster heads they are attached to based on the signal strength received. A hybrid, energy-efficient, distributed clustering algorithm (HEED) [5], a stable election algorithm (SEP) [6] and a hierarchical agglomerative clustering algorithm (DHAC) [7] have improved the LEACH algorithm. In recent years, the clustering method and the method of cluster head selection are optimized, and many cluster algorithm have been proposed. However, using multi-hop transmission method, nodes consume a lot of energy in data transmission. Additionally, in many environments, the battery cannot be replaced after the nodes’ power is exhausted.

References [8,9,10,11] have introduced mobile sink to collect data. In traditional methods, the sink is static, and waits to receive data packets generated by sensor nodes. In order to reduce the energy consumption for sensor nodes sending data to the sink, the mobile sink is used in some scenarios. The mobile sink can move to the vicinity of the nodes to collect data within the monitored range. Due to the constant movement of the sink, the nodes need to broadcast their own information and track the sink constantly, which also consumes a lot of energy. Therefore, many literatures propose the idea of using mobile collectors to collect data packets.

Reference [12] has proposed a data collection method based on the genetic algorithm. First, the nodes within the network are divided into several clusters. Then the overlaps of the communication ranges are found. Mobile collectors move to these locations to collect data packets, and an algorithm is proposed to calculate the paths for them. The defect of the algorithm is that the data packets are sent to the sink after all the data packets have been collected, so the data delay is long. When there are multiple mobile collectors in the network, the delay can be reduced compared to only one mobile collector. The goal of reference [13] is to plan the paths of multiple mobile collectors and minimize the data delay. There is a probability that a region is visited by different mobile collectors, which results in the waste of resources. The reference [14] has divided the data collection problem into three problems: task scheduling, path calculation, and speed control. The greedy algorithm is introduced to assign tasks to mobile collectors. The mobile collectors need to visit each node, therefore the data delay is long. The goal of reference [15] is to select the optimal collection point. First a node sets itself as the collection point. Then it sends information to neighbor nodes within its multi-hop range, including its identification, the number of neighbor nodes and the distance from the sink. When it finds a node closer to the sink and having more neighbor nodes than it, it marks the node as a collection point. This algorithm is suitable for the environment where nodes are evenly distributed. When nodes are randomly distributed, it is difficult to get the optimal solution. The problem solved by reference [16] is how to plan a path for each mobile collector. This problem is defined as an optimization problem and solved by a linear programming approach. Reference [17] considers a scenario where an unmanned aerial vehicle collects data from a set of sensors on a straight line. Reference [18] proposes a fault tolerant algorithm for data aggregation to plan the itinerary for a mobile agent and another alternative itinerary in case of sensor nodes failure.

In this paper, the wireless sensor network is distributed in the monitored area. The sensor nodes are event-driven. The problem to be solved in this paper is, how multiple mobile collectors collaborate to collect event data packets generated by sensor nodes with the minimum data delay.

Game theory studies the decision-making behavior when decision-makers interact with each other. Game theory researches how individuals make decisions and how they are influenced by other players. Therefore, each participant’s decision includes the decisions of others. In general, game theory attempts to open the black box of individual decision-making.

The innovations of this paper include the following aspects.

(1) The matching game model in game theory is introduced. The problem of cooperative data collection by multiple mobile collectors is modeled as the matching problem between mobile collectors and high-level cluster heads.
(2) According to the preferences of mobile collectors and high-level cluster heads, their benefit ranking matrices are built. Then, a one-to-one matching algorithm is proposed.
(3) Theoretical analysis proves that the matching result between mobile collectors and high-level cluster heads is stable, optimal and unique.

## 2. Data Collection Method Based on Matching Game Theory

### 2.1. Preliminaries

The following assumptions are made for our considered network environment in this paper.

(1) Sensor nodes are distributed in the area of interest randomly, and all the sensors are static.
(2) The positions of sensor nodes are obtained by GPS or existing positioning algorithms. All the sensor nodes have homogeneous capabilities, such as the sensing range, communication range, fusion power and ability of localization.
(3) The sensor nodes are event-driven. They generate event packets when they detect events of interest to the sink. Then the event packets are sent to the sink by mobile collectors. The sink is static.
(4) The network topology is connected.

Some definitions are given as follows.

Mobile collector: A mobile collector is a power unit which can move freely and carries radio frequency transceivers, which is expressed by $g_{i}$, where i is the identification of the mobile collector.

Visit: For any sensor node $S_{n}$, if a mobile collector communicates with it and receives data packets from it, it is called that $S_{n}$ is visited by the mobile collector.

High-level cluster head: In the three-layer sensor network, sensor nodes are separated to play different roles, such as cluster heads and ordinary nodes. First, all nodes are grouped into low-level clusters and the low-level cluster heads are elected by a cluster algorithm. Then, the low-level cluster heads are organized into high-level clusters and high-level cluster heads are elected. A high-level cluster head is expressed by $v_{j}$, where j is the identification of the high-level cluster head.

Request sequence set: During a collection cycle, the data packets generated by ordinary sensor nodes are relayed to high-level cluster heads through low-level cluster head. When a high-level cluster head has received these data packets, it will send a request message to the sink. The request message received by the sink during a collection cycle constitutes a set named RG, which is expressed as Equation (1), where $RG_{n}(t_{n},\varepsilon_{n},(x_{n},y_{n}))$ is the request information sent by the high-level cluster head named $v_{n}$, $t_{n}$ is the time when the data packets received by $v_{n}$ were generated, $\varepsilon_{n}$ is the priority of these data packets, $x_{n}$ and $y_{n}$ are the abscissa and ordinate of $v_{n}$, respectively. There are two cases. In one case, the generation times and priorities of the data packets received by $v_{n}$ are the same. Then the generation time and priority are the values of $t_{n}$ and $\varepsilon_{n}$. In another case, the generation times and priorities of the data packets received by $v_{n}$ are different. Then $t_{n}$ is defined as the minimum of these generation times, and $\varepsilon_{n}$ is defined as the maximum of these priorities.

(1) $$RG=\{RG_{a}(t_{a},\varepsilon_{a},(x_{a},y_{a})),RG_{b}(t_{b},\varepsilon_{b},(x_{b},y_{b})),\ldots ,RG_{k}(t_{k},\varepsilon_{k},(x_{k},y_{k}))\}$$

### 2.2. System Model

A wireless sensor network consists of many sensor nodes deployed in a monitored region. Each sensor node has a communication range $r_{c}$, which is a circle with the position of the node as the center and $r_{c}$ as the radius. Each sensor node consists of four components: sensing unit, microcontroller unit, radio unit, and a battery. The topology of the sensor network is represented by the graph Γ=(Κ,Ζ), where Κ represents the set of sensor nodes and Ζ represents the set of wireless links. For any two different nodes $S_{1},S_{2}\in Κ$, we say the wireless link $(S_{1},S_{2})\in Ζ$ if $d(S_{1},S_{2})\leq r_{c}$; otherwise $(S_{1},S_{2})\notin Ζ$, where $d(S_{1},S_{2})$ is the Euclidean distance between $S_{1}$ and $S_{2}$. In order to form a topology for a wireless sensor network, the medium access control (MAC) protocol proposed in reference [19], named self-organizing medium access control for sensor networks (SMACS), is used in this paper. By SMACS [19], the sensor nodes can discover their neighbors and establish transmission/reception schedules for communicating with them. After the topology of the wireless sensor network is formed, mobile collectors can communicate with sensor nodes. Since the sensor nodes use time division multiple access (TDMA) technology, some time slots are reserved for mobile collectors to send invitations. When a mobile collector needs to communicate with a node, it moves to the communication range of this node, sends an invitation to the node and waits for a reply from the node. If the node is unwilling to establish a connection with the collector, it sends a rejection message to the mobile collector. If the node accepts the invitation from the mobile collector, it sends a reply to the collector. Then the collector is registered at the node, and the node allocates some time slots for the communication between them. When the communication between them ends, the collector informs the node to release the connection.

The problem of data collection can be solved by the following steps.

(1) The wireless sensor network is grouped into three-layer clusters by a clustering algorithm.
(2) During a collection cycle, the request sequence set RG is sent to each collector by the sink. Based on the matching game theory, each collector selects a high-level cluster head.
(3) Each collector moves to the communication range of a high-level cluster head and visits it. Then the collector sends the data packets which it has received to the sink.

**Figure 1:**
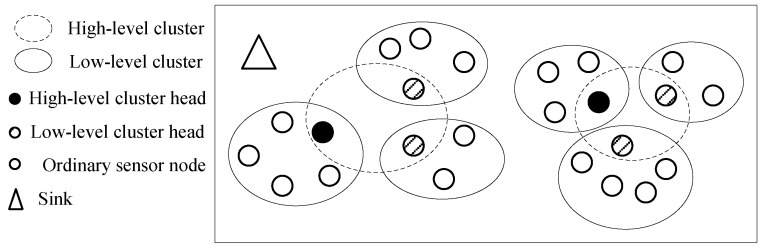
Architecture of the wireless sensor network.

The step 1 can be solved by a clustering algorithm. Many literatures have proposed clustering algorithms, therefore the clustering algorithm is not the focus of this paper. For example, the self-organization clustering algorithm (SOC) proposed in reference [20] can be used in this paper. Architecture of the wireless sensor network is given in Figure 1. In step 3, the collectors communicate with high-level cluster heads and the sink, and relay data packets from high-level cluster heads to the sink. According to the above analysis, steps 1 and 3 are relatively easy to implement, so the focus of this paper is step 2.

A system that applies a one-to-one matching game model must satisfy at least two important assumptions.

(1) From the beginning of the game, participants must belong to two disjoint sets. For example, the sets of the two sides are S and T, respectively, and satisfy Equation (2).
  (2) S∩T=∅
(2) A match can only be formed after the agreement of both sides.

A number of mobile collectors are randomly scattered in the monitored area. During each collection cycle, each collector selects a high-level cluster head to visit by a matching-game based algorithm. This process is accomplished by the communication between collectors and high-level cluster heads. Let $G=\{g_{1},g_{2},\cdots ,g_{n}\}$ and $V=\{v_{1},v_{2},\cdots ,v_{m}\}$ denote the set of collectors and the set of high-level cluster heads which have received data packets from low-level clusters, respectively. Thus, their relationship satisfies Equation (3). The benefit matrices of the two sets G and V are represented by $C_{M\times N}$ and $H_{M\times N}$, respectively, where N and M represent the number of elements in G and V, respectively. G and V make decisions based on $H_{M\times N}$ and $C_{M\times N}$.

(3) G∩V=∅

Suppose the symbol ≻ represents a strict preference relationship. For G, x, y, z represent three options. If G prefers x to y, it can be expressed as x≻y. For G, the following three conclusions hold.

(1) The expression x≻y holds or the expression y≻x holds. Thus ≻ is complete.
(2) If the expressions x≻y and y≻z hold, the expression x≻z holds. Thus ≻ is transferable.
(3) ≻ is complete and transferable, therefore ≻ is rational.

Similarly, the preference of V is rational. Therefore, G and V are two disjoint, rational sets. Furthermore, the problem studied in this paper can be modeled as a one-to-one matching problem between collectors and high-level cluster heads which have received data packets from low-level clusters.

Each mobile collector ranks the high-level cluster heads in the order of its preference. Suppose that the set of preferences of a mobile collector $g_{n}$ on the set V is represented by $P(g_{n})$. For example, Equation (4) shows that, $g_{n}$ ranks $v_{1}$ first, $v_{5}$ second, $v_{8}$ the last. This indicates that $g_{n}$ most wants to match $v_{1}$, followed by $v_{5}$, and finally $v_{8}$, which can be expressed as $v_{1}{≻}_{g_{n}}v_{5}$, $v_{5}{≻}_{g_{n}}v_{8}$. Similarly, the set of preferences of a high-level cluster head $v_{n}$ on the set G is represented by $P(v_{n})$. Equation (5) shows that, $v_{n}$ ranks $g_{1}$ first, $g_{3}$ second, $g_{10}$ the last. The preference set of two sides is represented by P. As shown in Equation (6), a two-sided matching market is expressed as (G,V;P).

(4) $$P(g_{n})=v_{1},v_{5},\ldots ,v_{8}$$

(5) $$P(v_{n})=g_{1},g_{3},\ldots ,g_{10}$$

(6) $$P=\{P(g_{1}),P(g_{2}),\ldots ,P(g_{n});P(v_{1}),P(v_{2}),\ldots ,P(v_{m})\}$$

**Theorem** **1.** *The matching function*μ:(G∪V)→(G∪V)*: If*μ(g)≠g*, then*μ(g)∈V*. If*μ(v)≠v*, then*μ(v)∈G. μ(x)*is called the matching object of*x*. The set of matched elements is represented by*μ(G,V).

**Theorem** **2.** *The kernel of a matching game is equal to the set of stable matches*.

When researching the matching game, one critical assumption is that the matching process is voluntary. An element can send a matching invitation to another element. It also can reject a matching invitation from another element. If a match process is not implemented, it is said to be blocked. This assumption determines the core problem of the matching process between G and V: the stability of the matching process.

If a match μ is upset by another match $\tilde{\mu }(g,v)$, $\tilde{\mu }$ is better than μ and μ is unstable. Therefore, if μ is not in the kernel, it must be blocked by another match $\tilde{\mu }$. For any g∈G, if the expression $\tilde{\mu }(g)\in V$ is valid, $\tilde{\mu }(g)$ is better than μ(g). Meanwhile, suppose the expression $\tilde{\mu }(g)=v$ is valid. Then v considers g to be better than μ(v). In brief, matches that are not in the kernel must be unstable, and unstable matches must not be in the kernel.

Therefore, the problem studied in this paper is equivalent to finding a stable matching set for the bilateral matching problem (G,V;P).

### 2.3. Benefit Matrix

The stable matching set of (G,V;P) is related to the benefit matrices of high-level cluster heads $H_{M\times N}$ and mobile collectors $C_{M\times N}$, which is expressed by Equations (7) and (8), where M is the number of high-level cluster head, and N is the number of collectors. The calculation method of $H_{M\times N}$ and $C_{M\times N}$ is introduced in this section.

(7) $$H_{M\times N}=\left[\begin{array}{cccc} h_{11} & h_{12} & \cdots & h_{1N}\\ h_{21} & h_{22} & \cdots & h_{2N}\\ \vdots & \vdots & \ddots & \vdots \\ h_{M1} & h_{M2} & \cdots & h_{MN} \end{array}\right]$$

(8) $$C_{M\times N}=\left[\begin{array}{cccc} c_{11} & c_{21} & \cdots & c_{M1}\\ c_{12} & c_{22} & \cdots & c_{M2}\\ \vdots & \vdots & \ddots & \vdots \\ c_{1N} & c_{2N} & \cdots & c_{MN} \end{array}\right]$$

Reference [6] has proposed the energy consumption model of sensor nodes. When the transmitter sends L-bit information to the receiver with a distance of d, the energy consumed is expressed by Equation (9). Where $\delta_{fs}$ is the amplification coefficient, and $\delta_{mp}$ is the multipath fading coefficient. The radio dissipates $\chi_{elec}$ per bit to run the radio circuitry. We find that, the farther the sender and receiver are, the more energy they consume while communicating. In addition, each sensor node has a fixed communication range. When a collector wants to communicate with a cluster head, if it is not within the communication range of the cluster head, it needs to move to the range first. The farther it is from the cluster head, the longer it needs to move, and the greater the data delay. If it is within the communication range of the cluster head, it can communicate with the cluster head without moving. The closer they are, the less energy the cluster head consumes when communicating with it. Therefore, no matter from the perspective of energy consumption or data delay, the benefit of a high-level cluster head is mainly affected by the distance to the matched collector. If a high-level cluster head $v_{m}$ selects a collector $g_{i}$, the benefit of $v_{m}$ is represented by Equation (10), where $d_{im}$ is the distance between $g_{i}$ and $v_{m}$, and θ is the weight of the distance factor.

(9) $$T_{x}(L,d)=\{\begin{array}{c} \begin{array}{cc} L\chi_{elec}+L\delta_{fs}d^{2}, & d\leq d_{0} \end{array}\\ \begin{array}{cc} L\chi_{elec}+L\delta_{mp}d^{4}, & d>d_{0} \end{array} \end{array}$$

(10) hmi=θdmi

When a collector selects a high-level cluster head to visit, it considers the following three factors: (1) the time when the data packet was generated; (2) the priority of the data packet; (3) the distance between them. If a collector $g_{n}$ selects a high-level cluster head $v_{j}$, the benefit of $g_{n}$ is represented by Equation (11), where $t_{j}$, $\varepsilon_{j}$, $\left(x_{j},y_{j}\right)$ can be obtained by querying $RG_{j}(t_{j},\varepsilon_{j},(x_{j},y_{j}))$ in request sequence set RG, $d_{jn}$ is the distance between $g_{n}$ and $v_{j}$ and α, β, γ are weights of the three factors.

(11) $$c_{jn}=\frac{\alpha }{t_{j}}+\frac{\beta }{d_{jn}}+\gamma \varepsilon_{j}$$

Analytic hierarchy process (AHP) is a mathematical-based technology to derive the deciding factors for complex problems [21]. According to AHP, the decision maker indicates the strength of preference by the pairwise comparison between deciding factors. By answering the two questions “Which of the two is more important?” and “By how much?” the pairwise comparison is finished [22]. In this paper, the pairwise comparison results are expressed by a matrix Q in Equation (12).

(12) $$Q=\left[\begin{array}{ccc} q_{11} & q_{12} & q_{13}\\ q_{21} & q_{22} & q_{23}\\ q_{31} & q_{32} & q_{33} \end{array}\right]=\left[\begin{array}{ccc} \alpha /\alpha & \alpha /\beta & \alpha /\gamma \\ \beta /\alpha & \beta /\beta & \beta /\gamma \\ \gamma /\alpha & \gamma /\beta & \gamma /\gamma \end{array}\right]$$

The square matrix Q satisfies the Equation (13), where λ is characteristic value, and the nonzero vector z is called the eigenvector of Q corresponding to λ. Constructing a weight vector L contains three elements of α, β, γ, as shown in Equation (14). L satisfies the Equation (15), which is proved in Equation (16). Therefore, the weights of the three factors can be obtained by computing the eigenvector of Q when the characteristic value λ is equal to 3. That is, to solve the homogeneous linear Equation (17), where E is the unit matrix.

(13) Qz=λz

(14) $${\left(L\right)}^{T}=[\begin{array}{ccccc} \alpha & & \beta & & \gamma \end{array}]$$

(15) QL=3L

(16) $$\left[\begin{array}{ccc} \alpha /\alpha & \alpha /\beta & \alpha /\gamma \\ \beta /\alpha & \beta /\beta & \beta /\gamma \\ \gamma /\alpha & \gamma /\beta & \gamma /\gamma \end{array}\right]\left[\begin{array}{c} \alpha \\ \beta \\ \gamma \end{array}\right]=\left[\begin{array}{c} 3\alpha \\ 3\beta \\ 3\gamma \end{array}\right]=3\left[\begin{array}{c} \alpha \\ \beta \\ \gamma \end{array}\right]$$

(17) (Q−3E)L=0

### 2.4. The One-to-One Matching Algorithm

After the benefit matrices are built, a one-to-one matching algorithm is proposed in this section. Some definitions are given as follows.

**Theorem** **3.** φ(i)*: The identification of the high-level cluster head which pre-matches with*$g_{i}$;

**Theorem** **4.** ϕ(j): *The identification of the collector which pre-matches with*$v_{j}$;

**Theorem** **5.** D(i): *The matching state vector of*$g_{i}$;

**Theorem** **6.** R(j): *The matching state vector of*$v_{j}$;

**Theorem** **7.** m(i): *A pointer to an element in*$C_{1\times M}^{i}$.

The steps of the one-to-one matching algorithm are as follows.

Step 1: During each collection cycle, for any collector $g_{i}\in G$, it computes the benefit matrix $C_{1\times M}^{i}$, which is expressed as Equation (18), where $c_{Mi}$ is defined by Equation (11). There is a special case. If a high-level cluster head has not received any data packet, the benefit for $g_{i}$ selecting it is equal to 0. For any high-level cluster head $v_{j}\in V$, it computes the benefit matrix $H_{1\times N}^{j}$, which is expressed as Equation (19), where $h_{jN}$ is defined by Equation (10). There is also a special case. If a high-level cluster head has not received any data packet, the benefit for it selecting any collector is equal to 0.

(18) $$C_{1\times M}^{i}=[\begin{array}{cc} c_{1i} & c_{2i}\begin{array}{cc} \cdots & c_{Mi} \end{array} \end{array}]$$

(19) $$H_{1\times N}^{j}=[\begin{array}{cc} h_{j1} & h_{j2}\begin{array}{cc} \cdots & h_{jN} \end{array} \end{array}]$$

Step 2: For any collector $g_{i}\in G$, it arranges the elements in $C_{1\times M}^{i}$ in descending order according to their values. Then the high-level cluster heads are sorted according to the arrangement result, which is represented by Equation (20). That is, $e_{1}$ represents the high-level cluster head which benefits $g_{i}$ the most, $e_{2}$ represents the second, and so on. Initially, $g_{i}$ sends invitations to the high-level cluster head represented by $e_{1}$.

(20) $$E=[\begin{array}{cc} \begin{array}{cc} \begin{array}{cc} e_{1} & e_{2} \end{array} & \cdots \end{array} & e_{M} \end{array}]$$

Step 3: Assuming the high-level cluster head which receives the invitation from $g_{i}$ is $v_{j}$. The following situations are discussed.

(1) If $v_{j}$ has not pre-matched any collector, R(j) satisfies the Equation (21). The following two situations are discussed.

(21) R(j)=0

(*i*) At the same time, if $v_{j}$ only receives the invitation from $g_{i}$, $v_{j}$ accepts this invitation. Then, R(j), D(i), ϕ(j) and φ(i) are expressed by Equations (22)–(25), respectively.

(22) R(j)=1v

(23) D(i)=1

(24) ϕ(j)=i

(25) φ(i)=j

(*ii*) At the same time, if $v_{j}$ receives invitations from multiple collectors, it inquires about $H_{1\times N}^{j}$, and selects the collector which benefits $v_{j}$ the most. If this collector is $g_{i}$, $v_{j}$ accepts the invitation from $g_{i}$. Otherwise, if this collector is not $g_{i}$, assuming $g_{a}$, $v_{j}$ accepts the invitation from $g_{a}$, and refuses the invitations from other collectors. After $g_{i}$ is refused by $v_{j}$, it, in turn, invites the cluster heads arranged behind $v_{j}$ in E. Then repeat step 3 until each cluster head has pre-matched with a collector.

(2) If $v_{j}$ has been pre-matched a collector, assuming $g_{b}$, R(j) and ϕ(j) must satisfy the Equations (22) and (26). The following two situations are discussed.

(26) ϕ(j)=b

(*i*) if $h_{jb}$ and $h_{ji}$ satisfy Equation (27), $v_{j}$ notifies $g_{b}$ to cancel the original pre-match $\mu (g_{b},v_{j})$, and accepts the invitation from $g_{i}$. Then the pre-match $\mu (g_{i},v_{j})$ is established, and D(b) is expressed by Equation (28).

(27) $$h_{jb}<h_{ji}$$

(28) D(b)=0

(*ii*) If $h_{jb}$ and $h_{ji}$ satisfy Equation (29), $v_{j}$ refuses the invitations from $g_{i}$. Then $g_{i}$ invites the cluster heads arranged behind $v_{j}$ in E in turn. Then repeat step 3 until each cluster head has pre-matched a collector.

(29) $$h_{jb}>h_{ji}$$

THEOREM 1 According to the matching algorithm proposed in this paper, there must exist a set of stable matches for the matching problem between G and V, and the matching result is stable, optimal and unique.

**Proof.**  According to the matching algorithm proposed in this paper, initially, each collector sends invitations to the high-level cluster head which benefits it the most. If a cluster head receives invitations from multiple collectors, it selects one collector which benefits it the most to accept its invitation, and rejects other collectors. Then these rejected collectors send invitations to their second preferred cluster heads. The algorithm terminates when all cluster heads are matched. Since the number of cluster heads is limited and the number of collectors is greater than or equal to the number of cluster heads, each cluster head must be able to find a collector to match it. Additionally, since the matching process is voluntary, the matching result must be consistent with the individual rationality. □

The next step is to prove that the matching result μ obtained by the matching algorithm is stable. Suppose $g_{i}$ and $v_{i}$ satisfy Equations (30) and (31). $g_{i}$ and $v_{i}$ prefer to form a match. That is, the Equations (32) and (33) are satisfied. Thus $g_{i}$ will send an invitation to $v_{i}$. The following two situations are discussed. (*i*) if $v_{i}$ has not pre-matched any collector, $v_{i}$ will accept the invitation from $g_{i}$. If it receives an invitation from $\mu (v_{i})$ later, it will refuse this invitation. (*ii*) if $v_{i}$ has had a pre-matching collector, $v_{i}$ will refuse this original pre-matching object and pre-match $g_{i}$. $g_{i}$ and $v_{i}$ have not formed a matching pair until the matching algorithm is finished. Thus $v_{i}$ must refuse the invitation from $g_{i}$, and the match $\left(g_{i},v_{i}\right)$ cannot block μ. Therefore, the matching result μ is stable.

(30) $$\mu (g_{i})\neq v_{i}$$

(31) $$\mu (v_{i})\neq g_{i}$$

(32) $$g_{i}{≻}_{v_{i}}\mu (v_{i})$$

(33) $$v_{i}{≻}_{g_{i}}\mu (g_{i})$$

Then prove that the matching result μ is optimal. Suppose there is another matching result $\tilde{\mu }$ better than μ. Then there is at least one cluster head satisfying the inequality (34), and $\left(\tilde{\mu }(v_{i}),v_{i}\right)$ must block μ. So μ is unstable. As previously proved, μ is stable. Hence this assumption does not exist, and μ is optimal.

(34) $$\tilde{\mu }(v_{i}){≻}_{v_{i}}\mu (v_{i})$$

Finally, prove that the matching result μ is unique. As previously proved, μ is optimal. Suppose there is another matching result $\tilde{\mu }$, and $\tilde{\mu }$ is another optimal match different from μ. Then there is at least one cluster head $v_{i}$ satisfying the inequality in Equation (35). Since μ is optimal, the inequality in Equation (36) holds. Since $\tilde{\mu }$ is optimal, the inequality in Equation (34) holds. Since ≻ means strict preference, the relationship between $\tilde{\mu }(v_{i})$ and $\mu (v_{i})$ must satisfy Equation (37). This conclusion contradicts the assumption that $\tilde{\mu }$ is different from μ. Therefore, μ is unique.

(35) $$\tilde{\mu }(v_{i})\neq \mu (v_{i})$$

(36) $$\mu (v_{i}){≻}_{v_{i}}\tilde{\mu }(v_{i})$$

(37) $$\tilde{\mu }(v_{i})=\mu (v_{i})$$

In conclusion, this matching result μ obtained by the matching algorithm proposed in this paper is stable, optimal and unique.

The pseudo code of the matching algorithm is given as follows.
**Algorithm**: The one-to-one matching algorithm1 for  $g_{i}\in G$   do 2    $C_{1\times M}^{i}=[\begin{array}{cc} c_{1i} & c_{2i}\begin{array}{cc} \cdots & c_{Mi} \end{array} \end{array}]$
3     $E=[\begin{array}{cc} \begin{array}{cc} \begin{array}{cc} e_{1} & e_{2} \end{array} & \cdots \end{array} & e_{M} \end{array}]$
4     $\mathrm{sort}(C_{1\times M}^{i},’descend’)=E$
5       D(i)=0 6 m(i)=1 7  end for 8 for $v_{j}\in V$   do 9    $H_{1\times N}^{j}=[\begin{array}{cc} h_{j1} & h_{j2}\begin{array}{cc} \cdots & h_{jN} \end{array} \end{array}]$
10 R(j)=0 11  end for 12 n=0 13 while n<M do 14    for $g_{i}\in G$ do 15      if  D(i)=0 then 16       $g_{i}$ invite the cluster head $v_{j}$ 17         if R(j)=0 then 18           φ(i)=j
19           ϕ(j)=i 20           D(i)=1 21           R(j)=1 22           n=n+1 23           m(i)=1 24         else 25           if $h_{jb}>h_{ji}$ then 26            if m(i)<M
27             D(i)=0 28             m(i)=m(i)+1 29            else 30             $g_{i}$ doesn’t invite any cluster head 31          else 32           φ(i)=j 33           D(b)=0 34           D(i)=1 35           m(i)=1 36     else 37       $g_{i}$ doesn’t invite any cluster head 38           end if 39           end if 40         end if 41       end if 42   end for 43  end while

## 3. Simulation Results

The performance of the matching algorithm proposed in this paper is evaluated by simulations in this section. In our simulations, sensor nodes are distributed uniformly in a square field without obstacles. The sink is located in the center of the area. Sensor nodes are event triggered. The parameter values used in the simulation are listed in Table 1. The simulations are performed using MATLAB R2017a. The communication range of each sensor node is set to 30 m. When a collector needs to communicate with a cluster head, if it is not within the communication range of the cluster head, it moves to the range first. Then it stops and sends an invitation message to the cluster head. If the cluster head accepts the invitation, a connection will be established between them. Then the cluster head stars sending data packets to the collector. After the collector has received the data packets, it notifies the cluster head to release the connection. Then the collector moves to another collection point. The matching game-based data collection algorithm for wireless sensor networks proposed in this paper is called MGDC. The matching game-based data collection algorithm (MGDC) is compared with the region based data collection algorithm (SOC-RDC) and the time based data collection algorithm (SOC-TDC) proposed in [20].

**Figure 2:**
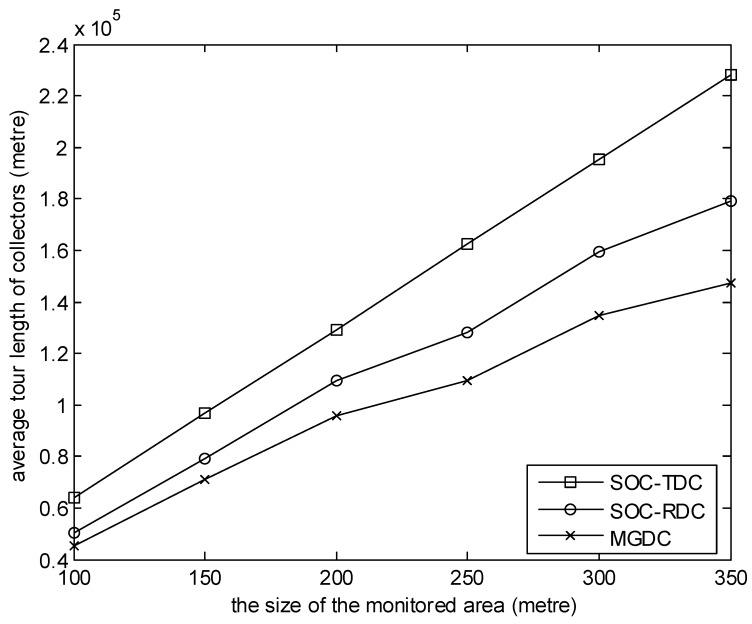
The size of the monitored area versus average tour length of collectors.

**Figure 3:**
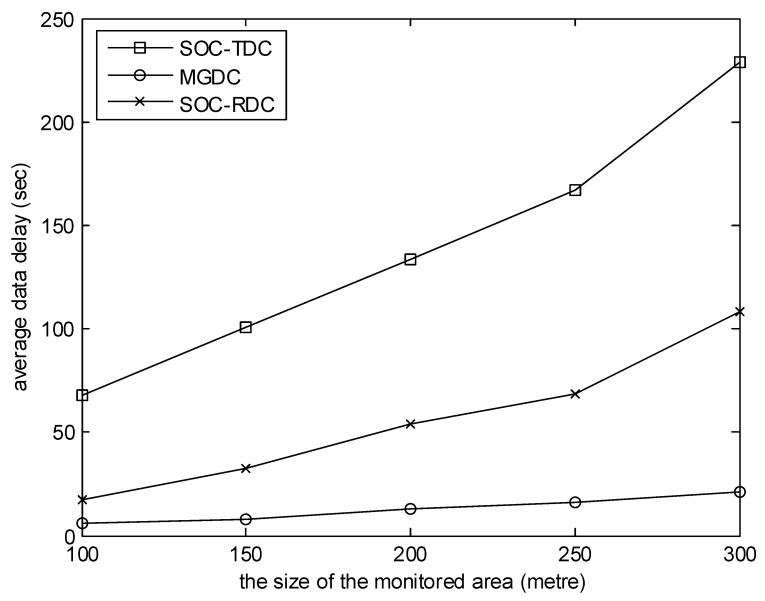
The size of the monitored area versus average data delay.

**Figure 4:**
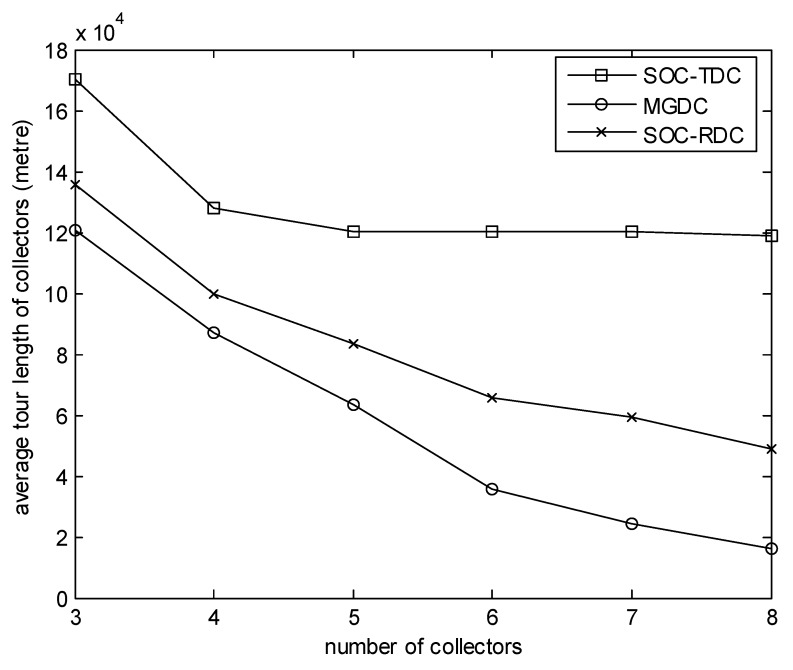
Number of collectors versus average tour length of collectors.

**Figure 5:**
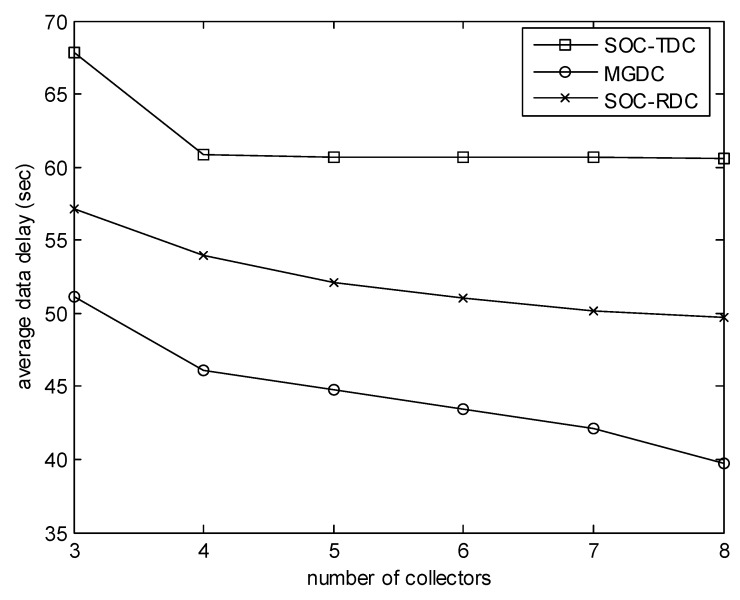
Number of collectors versus average data delay.

**Figure 6:**
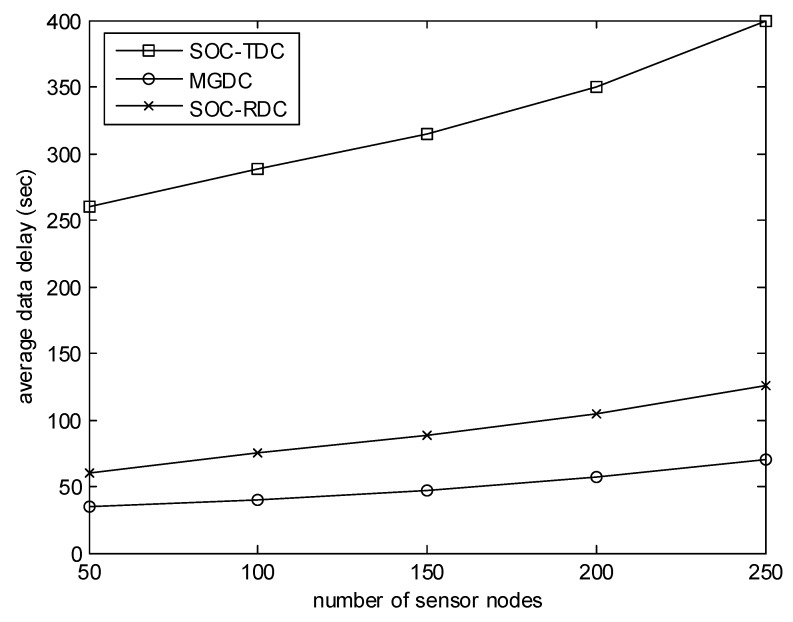
Number of sensor nodes versus average data delay.

In the simulations shown in Figure 2, Figure 3, Figure 4, Figure 5 and Figure 6, Equation (38) is satisfied. Figure 2 shows the relationship between the average tour length of collectors and the size of the monitored area during the simulation time. In the simulation, the number of collectors is 8, and the number of sensor nodes is 100. It can be observed from the figure that, with MGDC, the average tour length of collectors is smaller than that of SOC-RDC and SOC-TDC, and with the increase of the size of the monitored area, the gap between them increases. There are two reasons for this result. One reason is that, using SOC-TDC, the data packets generated at the same time are collected by one collector. These data packets may be scattered over different regions, so the collector moves a long distance to collect them. According to the MGDC, during a collection cycle, a collector only visit a high-level cluster head, so it moves a short distance. Another reason is, according to SOC-RDC, data packets generated at the same time are collected by several collectors, not one collector. Some collectors may select the same region. Under this circumstance, the sink randomly selects one of these collectors to collect data packets in this region. The choice may not be optimal. However, the matching result of MGDC between collectors and high level cluster heads is optimal. With the increase of the size of the monitored area, the distribution of sensor nodes is becoming more and more dispersed, so the advantages of MGDC are becoming more and more obvious.

(38) α:β:γ=1:1:1

Figure 3 shows the relationship between the average data delay and the size of the monitored area during the simulation time. Data delay is the time difference between the data packet generated and the data packet collected by a collector. In the simulation, the number of collectors is 8, and the number of sensor nodes is 100. It can be seen from the figure that the average data delay of MGDC is smaller than that of SOC-RDC and SOC-TDC. And as the size of the monitored area increases, the gap between them increases. One reason is that, using MGDC, the average tour length of collectors is smaller than that of SOC-RDC and SOC-TDC. Another reason is that, using MGDC, the matching process between collectors and cluster heads takes into account several factors, such as time and position, rather than considering a single factor.

Figure 4 focuses on the effect of the number of collectors on the average tour length of collectors. In this experiment, the number of sensors is 100, and the size of the monitored area is 100 m. We can observe that when the number of collectors is small, the advantage of MGDC is not obvious. This is because, during the simulation time, when the number of high-level cluster heads which have received data packets is greater than the number of collectors, one collector needs to visit several cluster heads. The average tour length of collectors is long. Furthermore, when the number of collectors is small, the options of cluster heads are few, and the difference between SOC-RDC and MGDC is not obvious. However, when the number of collectors increases, the combinations between cluster heads and collectors increase, and the advantages of MGDC become more obvious. Since the data packets generated at the same time are collected by one collector according to SOC-TDC, as the number of collectors increases, some collectors are idle. Hence, for SOC-TDC, as more collectors are added, the impact on average tour length of collectors is not significant.

Figure 5 highlights the effect of the number of collectors on the average data delay. In this experiment, the number of sensors is 50, and the size of the monitored area is 100 m. We can find that MGDC performs better than SOC-RDC and SOC-TDC. The more the number of collectors, the more obvious the advantage of MGDC.

Figure 6 evaluates the effect of the number of sensor nodes on the average data delay. In this experiment, the number of collectors is 6, and the size of the monitored area is 400 m. As illustrated in Figure 6, when the number of sensor nodes increases, the average data delay increases. The reason is that, when the number of nodes is small, the number of high-level cluster heads is small, and there are few optional objects for collectors. The gap between MGDC and SOC-RDC is not obvious. With the increase of the number of sensor nodes, the number of clusters and nodes which have monitored events increases. There are more optional objects for collectors, and the gap between MGDC and SOC-RDC becomes more and more obvious.

**Figure 7:**
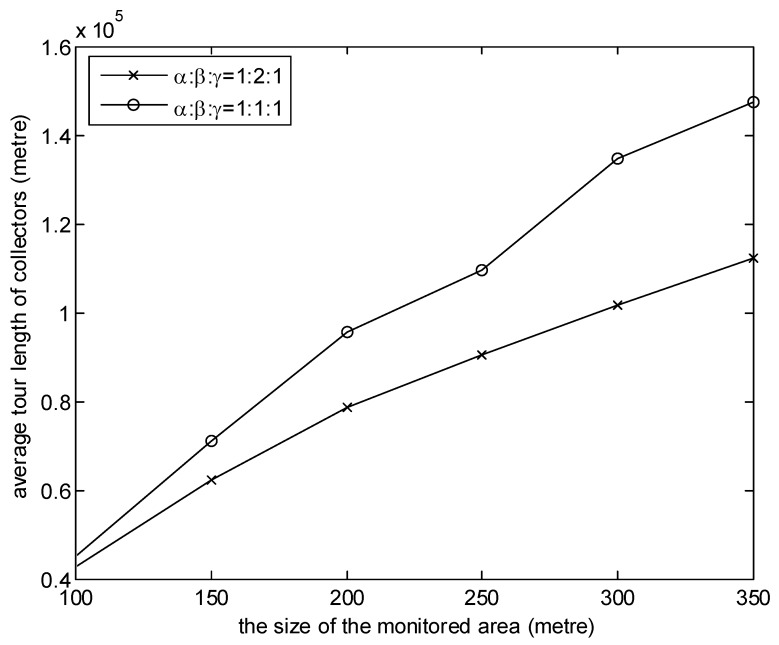
The effect of the weights on average tour length of collectors.

**Figure 8:**
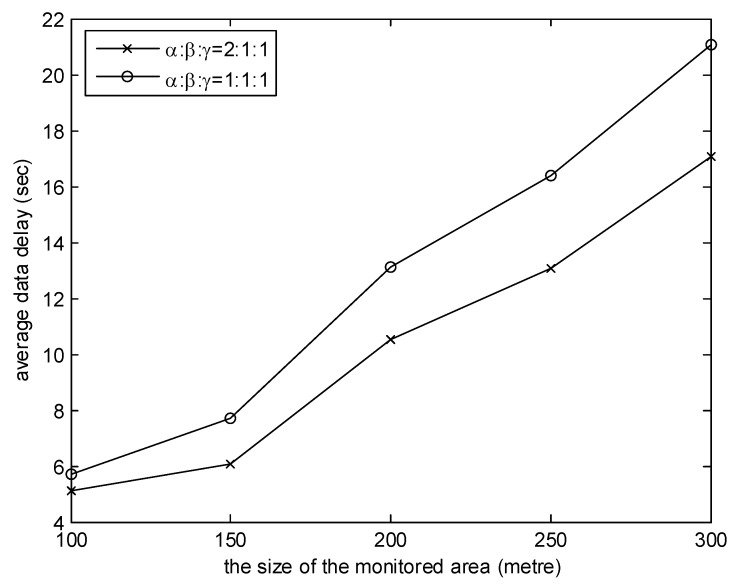
The effect of the weights on average data delay.

Figure 7 and Figure 8 investigate the effects of the weights of three factors of MGDC. In the simulations, the number of collectors is 8. It is observed from the graph in Figure 7 that the higher the proportion of β, the shorter the average moving distance. This is because, when the proportion of β is high, according to the benefit of the collector obtained from Equation (11), collectors will give priority to the near cluster head. As illustrated in Figure 8, the higher the proportion of α, the smaller the data delay. The reason is that, when the proportion of α is high, according to the benefit of the collector obtained from Equation (11), the cluster head which has collected the data packets with the earlier generation time will be the first choice of collectors.

## 4. Conclusions

Sensor nodes consume most of energy for data transmission and the battery of sensor nodes cannot be replaced in many complex environments, therefore it is necessary to introduce mobile collectors to collect data. Since the speed of collectors is limited, it will cause data delay. Therefore, the problem of this paper is how to make multiple collectors cooperate with each other to reduce data delay. Based on the matching game theory, the problem is modeled as a one-to-one matching problem between collectors and high-level cluster heads. Then, a one-to-one matching algorithm is proposed. Simulation results show that as the size of the monitored area, the number of collectors and the number of sensors increase, the advantage of this algorithm in reducing data delay becomes more and more obvious.

## Figures and Tables

**Table 1:** Simulation parameters.

Parameters	Values
radio bandwidth of sink (Mbps)	1
sensing range of a sensor node (m)	20
transmission range of a mobile collector (m)	35
speed of a mobile collector (m/s)	5
event packet size (bytes)	100
request packet size (bytes)	25
packet head size (bytes)	25
simulation time (s)	40,000
